# Ward-Specific Probabilistic Patterns in Temporal Dynamics of Nursing Demand in Japanese Large University Hospital: Implication for Forecasting and Resource Allocation

**DOI:** 10.1155/2024/2554273

**Published:** 2024-07-09

**Authors:** Rie Tajika, Yoshiaki Inoue, Keisuke Nakashima, Takako Yoshimi, Nobue Arimoto, Haruna Fukushige, Yoko Taniura, Tomoyuki Iwasaki, Atsue Ishii

**Affiliations:** ^1^ Department of Nursing Graduate School of Health Sciences Kobe University, 7-10-2 Tomogaoka, Suma-ku, Kobe 654-0142, Hyogo, Japan; ^2^ Department of Information and Communications Technology Graduate School of Engineering Osaka University, 2-1 Yamadaoka Suita, Osaka 565-0871, Japan; ^3^ Department of Medical Informatics Graduate School of Medicine Osaka University, 2-2 Yamadaoka Suita, Osaka 565-0871, Japan; ^4^ Division of Nursing Osaka University Hospital, 2-15 Yamadaoka Suita, Osaka 565-0871, Japan

## Abstract

As global populations age, a looming nursing shortage is anticipated to become a critical issue. Charge nurses have the responsibility of optimally allocating nursing resources to ensure the quality of patient care during a shift. Therefore, an accurate estimate of nursing demand is crucial. However, the ability to forecast future nursing demand remains underdeveloped, mainly because the nature of nursing demand is highly individualized and does not follow a definitive pattern. In practice, the nursing demand is often perceived as unpredictable, leading to an ad hoc approach to staffing. The primary objective of our study is to demonstrate that longitudinal data analysis can reveal strong statistical regularities in the temporal dynamics of nursing demand. This approach not only provides new possibilities for efficient resource allocation but also paves the way for data-driven prediction of nursing demand. Our study uses Sankey diagrams to visualize the temporal dynamics of nursing demand within each ward for each fiscal year, representing these dynamics as an overlay of trajectories from multiple individual patients. Consequently, our study reveals ward-specific statistical regularities in the temporal dynamics of nursing demand. In one ward, approximately 25% of patients experienced an increase in nursing demand from 1 to between 6 and 9 points from the second to the third day of hospitalization, while in another, only 0.1% showed such an increase. These findings suggest that patients admitted to the wards tend to exhibit a certain probabilistic change in nursing demand. This study can predict probabilistically the temporal variation of nursing demand among patients in the coming years by analyzing data on the temporal changes in nursing demand over the past years. Our findings are expected to significantly influence the forecasting of nursing demand and the estimation of nursing resources, leading to data-driven and more efficient nursing management.

## 1. Introduction

As societies globally progress towards a superaging demographic, a looming nursing shortage is anticipated to become a critical issue. While it is imperative to expand nursing education to augment medical resources, the onus is also on charge nurses, who lead their nursing units during a shift, to ensure the optimal allocation of these resources [1]. This is paramount to maintaining the necessary quality of patient care, as research shows that increasing a nursing workload often leads to worse patient outcomes [2–4]. However, challenges remain in applying these findings to clinical practice [5]. To achieve more realistic staffing, optimized nurse staffing has been examined, taking into account stakeholder decision-making involving nurses and patients [5, 6], as well as cost considerations [7]. Furthermore, a nonlinear staffing model has been proposed to address the complex relationships caused by path dependencies and feedback loops in staffing factors [8]. Among the complex factors, nursing demand is particularly important as a core factor due to its significant impact on various nursing resources [8]. Consequently, accurately estimating nursing demand emerges as a fundamental challenge that must be addressed.

Efforts to quantify nursing demand have been undertaken in several countries. The Nursing Activities Score (NAS) is utilized to measure both direct and indirect nursing activities in various countries [9, 10]. In Finland, the Oulu Patient Classification (OPC) is employed to evaluate the volume and intensity of nursing care provided to each patient [11, 12]. In Japan, the Intensity of Nursing Care Needs (INCN) serves as a measure of nursing demand. While progress has been made in quantifying current nursing demand, the ability to forecast and predict future demand remains under-researched.

A key challenge in estimating nursing demand lies in the highly individual nature of patient care demand [13]. Patients present with a wide array of physical and psychosocial backgrounds, leading to considerable variability in nursing demand. As a result, the daily nursing care required throughout a patient's hospital stay until discharge does not follow a simple, deterministic pattern. This variability persists even among patients hospitalized for similar diseases, as care demand can greatly differ from one individual to another. In practice, it is often assumed that nursing demand is inherently unpredictable, leading to an ad hoc approach in the allocation of nursing resources.

To address clinical challenges, many studies have attempted to predict nursing demand based on data. However, factors such as age [14, 15] and emergency situations [14] were not found to be associated with nursing demand. Instead, the relationship with sex varied depending on the situation [14, 15]. Furthermore, although some studies have reported that the length of stay and survival [15, 16] are useful variables, these factors cannot be incorporated into prospective prediction models. One study [13] suggested that nursing demand can help predict which patients will have stable nursing needs in the subsequent shift, but it did not address the ability to predict changes in nursing demand for patients. Less than half of the patients had stable nursing demand. Therefore, the development of a model applicable to all patients is ongoing. Nursing demand changes dynamically, which may complicate staff allocation based on nursing demand [13]. Consequently, it is essential to explore methods to predict dynamic nursing demand effectively.

The primary objective of this paper is to illustrate that longitudinal data analysis can reveal strong *statistical regularities* in the temporal dynamics of nursing demand. This serves as a preliminary step toward solving the nursing demand estimation challenge. Here, the term “statistical regularity” refers to consistent patterns within data that inherently contain randomness. In other words, it describes empirical phenomena where observations do not yield the same outcome each time yet show statistical stability in their frequency [17].

The influence of patient individuality, which contributes to the diversity of nursing demand, can be statistically encapsulated within these regularities when considering the entire patient population as a collective entity. Successfully identifying these statistical regularities paves the way for formulating nursing demand forecasting and resource allocation as a stochastic, or probabilistic problem. This approach not only opens up new possibilities for efficient resource allocation in nursing management but also provides a data-driven method to anticipate nursing demand despite the seeming unpredictability of individual patient nursing demand over the course of hospitalization days.

In this study, we analyzed three years' worth of data related to item “B” of the INCN from a large Japanese hospital with over a thousand beds. As will be detailed in the Methods section, item “B” of the INCN evaluates patient's activities of daily living (ADL) and cognition. Importantly, nurses often encounter physical strain when caring for patients with low ADL and cognition. Such care tasks typically involve manual handling of patients, including positioning and transfers, which directly contribute to the physical workload experienced by nurses [18–20].

To provide insights that can be useful for nursing staff scheduling [21] and bed management [22], our focus is on the temporal evolution of nursing demand throughout the entire hospitalization period. The key to uncovering statistical regularities from this perspective is the utilization of a visualization tool known as *Sankey diagrams*.

Sankey diagrams, a type of flow diagram, are widely used in fields such as industrial engineering, economics, and finance to visualize the magnitude of various flows. In medical fields, it has been used to investigate usage paths in mobile electronic health record systems [23] or to visualize treatment courses for a group of patients [24]. When the nodes in these diagrams are appropriately aligned, they can provide a clear and comprehensive picture of the dynamic changes in nursing demand throughout the course of a patient's hospital stay. Nurses in clinical practice sites encounter highly individualized patients, which can obscure the regularities inherent in the patient population. Their knowledge is often limited to the patients for whom they are responsible, restricting their ability to gain an overview of nursing demand trends across the entire patient population. However, visualizing nursing demand with Sankey diagrams enables observation of these trends for the entire patient population from a bird's eye view, which allows for capturing the statistical regularities in the nursing demand dynamics of the patient population. As demonstrated in the following sections, this approach presents an effective way to understand the evolution of nursing demand over time.

## 2. Materials and Methods

### 2.1. Design and Setting

**Figure 1:**
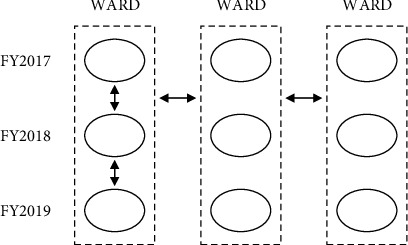
Overview of comparative analysis of patterns in the trajectories of nursing demand by fiscal year and ward. (1) Comparing the trajectories across fiscal years for each ward (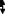). (2) Comparing the trajectories between different wards (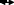).

With Sankey diagrams, we visualized statistical patterns in the trajectories of nursing demand for each ward for each fiscal year. We analyzed the results from the following two perspectives (Figure 1):

1. For each ward, we assessed the similarity/dissimilarity in the visualized trajectories of nursing demand across fiscal years
2. We compared the visualized trajectories of nursing demand between different wards

### 2.2. Target Hospital

This study focused on a large university hospital in Japan with over a thousand beds. As a university hospital, the targeted hospital serves multifaceted roles: it functions as an educational institute for fostering medical professionals, as a research institute for the innovation of novel medical technologies, and as a central regional healthcare institution offering advanced healthcare provisions. Consequently, the hospital is responsible for treating patients requiring advanced medical care. In the wards studied, the ratio of the number of patients to that of nurses is 7 : 1.

### 2.3. Data Collection

#### 2.3.1. Intensity of Nursing Care Needs

The INCN is an indicator used in Japan to measure the demand for nursing care. It comprises three components: items “A,” “B,” and “C.” Item “A” evaluates the expertise of nurses and encompasses subitems related to monitoring and treatment procedures, such as pressure ulcer care and syringe pump management. Item “B” assesses the level of caregiving for recuperation administered by nurses. It includes subitems that evaluate the patient's ADL and cognition. Item “C” appraises surgical procedures considered urgent from a medical standpoint. It is scored based on the type and volume of medical procedures undertaken, such as craniotomy or open chest surgery, and it is assessed within a specific time frame in postprocedure. Nurses evaluate the nursing demand for each patient every day. The electronic medical record stores the resulting INCN scores, thereby preserving the data as a time series.

In this study, we utilized the score for item “B” of the INCN of individual patients. As mentioned above, Item “B” evaluates a patient's ADL and cognition (Table 1). Each score is recorded once a day, reflecting the most severe condition observed between 00 : 00 and 23 : 59. For the “risk behavior,” if patients engage in such behaviors within the past seven days, nurses mark “display risk behavior” for the target date. Risk behaviors include self-extubation during treatment or examination, falls, self-injury, as well as other behaviors that would lead to dangerous actions if left unaddressed. The score of item “B” ranges from 0 to 12, with higher scores indicating the need for more extensive or diverse types of nursing care.

#### 2.3.2. Target Data

This study utilized daily scores of Iiem “B” in the INCN as an indicator of nursing demand. We focused on scores from the first day of hospitalization (hereafter referred to as day 1) to day 18 for each patient. This decision stemmed from the fact that inwards operating under the 7 : 1 patient-to-nurse ratio, the average patient stay is optimized to be within 18 days. This study population comprised patients who were admitted to the target hospital during any of the following periods: from April 1, 2017, to March 14, 2018; from April 1, 2018, to March 14, 2019; and from April 1, 2019, to March 14, 2020, reflecting the fact that fiscal years in Japan start at April 1. Furthermore, these patients had to occupy general beds in either the Orthopaedic Surgery Ward, the Neurosurgery Ward, or the Cardiovascular Medicine Ward for at least one day before day 18. We treated each admission date for the same patient as a separate case. We excluded patients with missing score data for at least one day between day 1 and day 18.

We chose the three wards categorized as musculoskeletal, neurology, and internal medicine departments. This selection was premised on the fact that the patients in these wards necessitated varied types of nursing care, aligning with their diverse diseases and corresponding treatment plans, which would result in different natures of nursing demand.

Many patients in the Orthopaedic Surgery Ward undergo procedures such as arthroplasty, tendon reconstruction, or tumor resection. In the Neurosurgery Ward, a large number of patients receive treatments such as craniotomy for brain tumors, vascular embolization, and stent placement. The Cardiovascular Medicine Ward sees many patients who require medication, cardiac catheterization, and in some cases, a heart transplant.

Along with the scores of item “B” in the INCN, we collected the following attributes for each record: the date, patient ID, admission date, discharge date, and details of the inpatient wards and beds occupied by the patient.

### 2.4. Ethical Considerations

Ethical approval for this study was granted by our hospital's Ethics Committee (approval no. 22251 (T2)). All participant data were anonymized to ensure the protection of their personal information. Information about the study was made available to potential participants via the hospital's website, providing the option for patients who stayed at the hospital during the study period to opt out if they wished.

### 2.5. Data Analysis

**Figure 2:**
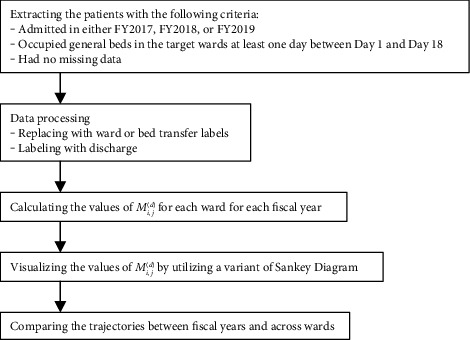
Flowchart for data analysis.

Figure 2 shows the flowchart of data analysis in our study. We collected data that met specific criteria, as mentioned previously in the Target Data section. We processed the data to account for patient transfers to other wards and discharges, which allowed for the analysis of data for each ward over the course of hospitalization. We calculated the number and proportion of patients who transitioned from one point to another each fiscal year in each ward. These trends were visualized using a type of Sankey diagram, which illustrates the flow and transition of scores among patients. We conducted a comparative analysis of the results from the Sankey diagrams and the number of patients within the same ward across different fiscal years. In addition, we compared the results from the Sankey diagrams and the proportion of patients across different wards.

In this analysis, Python version 3.11.7 served as the primary programming language [25]. We use Pandas 2.1.1 [26] and NumPy 1.25.0 [27] for the identification of the eligible patient population and the quantification of the total number of eligible patients and specific conditions within this population. Furthermore, we use Plotly 5.15.0 [28, 29] to create the Sankey diagrams.

#### 2.5.1. Data Processing

Each patient was assigned a daily score ranging from 0 to 12 points. Some patients were admitted to more than one ward or specialized beds such as Coronary Care Units during their hospital stay. If patients stayed in other wards or specialized beds, we replaced the scores with the label “staying in other wards or specialized beds.” For instance, when targeting a single ward and analyzing one patient, if this patient stayed in the target ward with a score of 0 points on day 1, in another ward or specialized bed with a score of 1 point on day 2, and back in the target ward with a score of 2 points on day 3, then we modified the sequence “0 points, 1 point, and 2 points” to “0 points, “staying in other wards or specialized beds,” and 2 points.” When a discharge was recorded, any data after the discharge record's entry date were labeled as “discharged.”

#### 2.5.2. Calculation

For each patient, the period of analysis was set to 18 days, as mentioned above, based on the average patient stay. Let *d* (*d* ∈ *𝒟*) denote the number of days elapsed after the admission to the hospital, where *𝒟*={1, 2,…, 17}. Let *ℰ* and *𝒮* denote the set of eligible patients and the set of daily scores including discharge, where *𝒮*={0, 1,…, 12} ∪ {staying in other wards or specialized beds, discharged}. For *e* ∈ *ℰ* and *i* ∈ *𝒮*, we define an indicator function *I*_*i*_(*e*, *d*) as follows:

(1) $$\begin{array}{c} I_{i}\left(e,d\right)=\left\{\begin{array}{cc} 1, & \text{if}\;\text{patient}\;e\;\text{scored}\;i\;\text{on}\;\text{Day}\;d,\\ 0, & \text{otherwise}. \end{array}\right. \end{array}$$

The total number *M*_*i*,*j*_^(*d*)^ of patients who scored *i* on day *d* and scored *j* on day *d*+1 is then given by

(2) $$\begin{array}{c} M_{i,j}^{\left(d\right)}=\underset{e\in E}{\sum }I_{i}\left(e,d\right)I_{j}\left(e,d+1\right). \end{array}$$

With *M*_*i*,*j*_^(*d*)^ and the total number *N*_e_ (*N*_e_=|*ℰ*|) of eligible patients, the proportion *P*_*i*,*j*_^(*d*)^ of patients who scored *i* on day *d* and scored *j* on day *d*+1 among eligible patients is calculated by

(3) $$\begin{array}{c} P_{i,j}^{\left(d\right)}=\frac{M_{i,j}^{\left(d\right)}}{N_{e}}. \end{array}$$

#### 2.5.3. Visualization

Sankey diagrams can visualize the flow of data between different stages. These diagrams clearly represent the distribution and transition of data points throughout the various phases by depicting the movement of quantities or information between numerous nodes in a system. The layout of the diagram is typically optimized and arranged automatically [30]. This method facilitates an intuitive understanding of the complex relationships and interactions within the dataset.

Unlike conventional automatically arranged Sankey diagrams, we arranged the vertical axis in descending order of scores for item “B,” while we aligned the horizontal axis with equal intervals to depict the elapsed days after hospitalization. Nodes are represented by rectangles indicating the score, and the flows between them are depicted by curves leading from source to target nodes. The volume of each flow, represented by the *M*_*i*,*j*_^(*d*)^, is indicated by the thickness of the curve. In other words, the curve's thickness corresponds to the number of patients whose scores transitioned from *i* on day *d* to *j* on day *d*+1. Note that, the Sankey diagram is composed of the volumes of flows between two days, which, in aggregation, also suggests typical trajectories of nursing demand. We visualized the values of *M*_*i*,*j*_^(*d*)^ for each ward for each fiscal year to investigate the statistical regularities in the temporal dynamics of nursing demand.

#### 2.5.4. Trajectory Comparison

The thickness of the flows corresponds to both the probability of the score transitioning and the volumes of flows. With the resulting Sankey diagrams and *M*_*i*,*j*_^(*d*)^ values, we compared the trajectories of the scores of the patient population between different fiscal years for each ward. Furthermore, we incorporated *P*_*i*,*j*_^(*d*)^ values alongside *M*_*i*,*j*_^(*d*)^ to compare the trajectories of the scores between different wards. Comparing the trajectories allows for the inference of the existence of probabilistic patterns in score transition.

## 3. Results

The basic characteristics of the target patients are shown in Table 2. In the Orthopaedic Surgery Ward, the eligible patient count was 684, 688, and 691 in the fiscal years 2017, 2018, and 2019, respectively. In the Neurosurgery Ward, the numbers were 839, 873, and 959 for those years, respectively. For the Cardiovascular Medicine Ward, there were 994, 998, and 898 patients in those years, respectively. The proportion of these patients was 99.3%. In the Orthopaedic Surgery Ward, the majority of patients were female, with a mean age in the mid-50s, consistently across three fiscal years. In the Neurosurgery Ward, the sex ratio was almost equal, with a mean age in the late 50s, also consistently across three fiscal years. In the Cardiovascular Medicine Ward, the majority of patients were male, with a mean age in the mid-60s, consistently over the same period. The median scores on day 1 were 1 point in the Orthopaedic Surgery Ward, almost 1 point in the Neurosurgery Ward, and 0 points in the Cardiovascular Ward, respectively.

The number of inpatients gradually decreased over the course of hospitalization in all wards, primarily due to patients being discharged as their hospital stays progressed. The nature of changes in scores varied across the different wards (Table 3).

We will present results from the following two perspectives:

1. The statistical regularities in temporal dynamics of nursing demand across fiscal years within each ward
2. The ward-specific feature of the statistical regularities in temporal dynamics of nursing demand

### 3.1. The Statistical Regularities in Temporal Dynamics of Nursing Demand across Fiscal Years within Each Ward

#### 3.1.1. The Orthopaedic Surgery Ward

**Figure 3:**
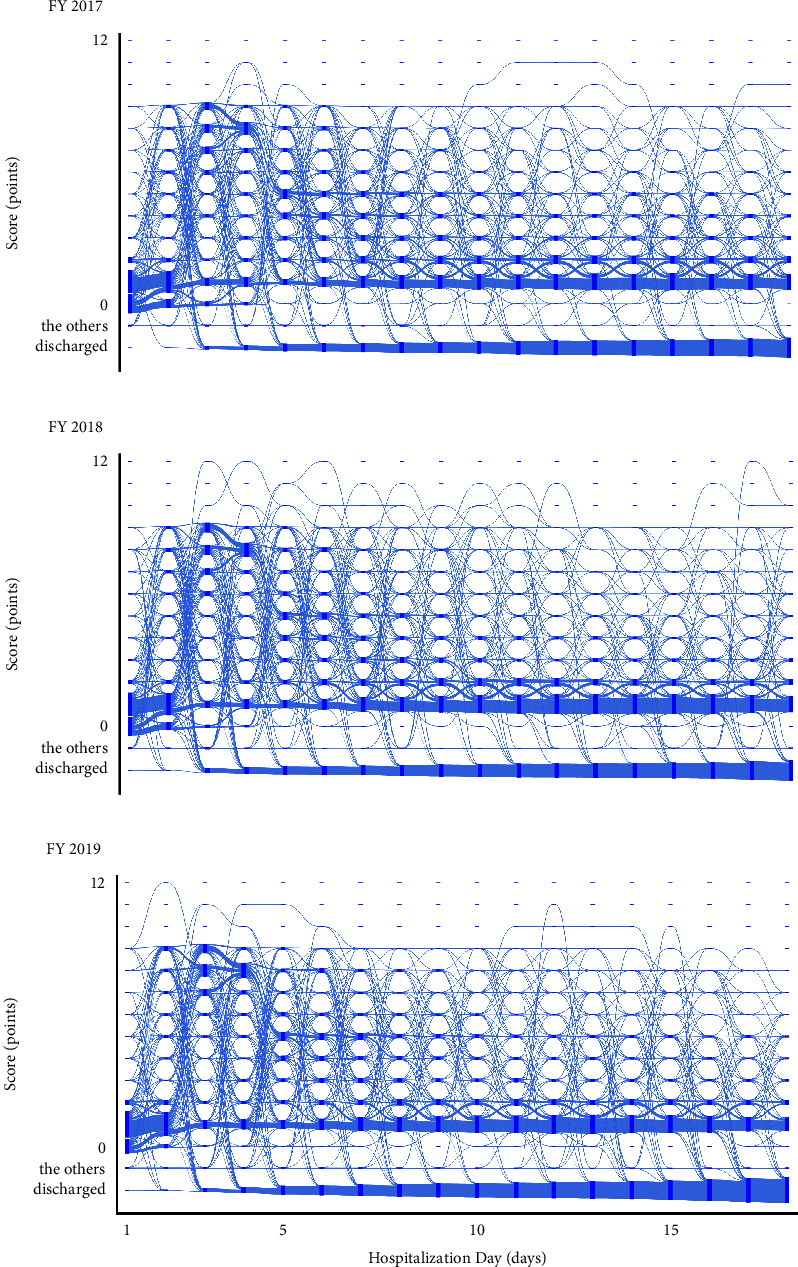
The trajectories of nursing demand in the Orthopaedic Surgery Ward for fiscal year (a) 2017, (b) 2018, and (c) 2019. Nodes are represented by rectangles indicating the score. The flows between them are depicted by curves leading from the source to the target nodes. The volume of each flow is indicated by the thickness of the curve. “The others” indicates “staying in other wards or specialized beds.”

All trajectories of the Orthopaedic Surgery Ward are shown in Figure 3. The *x*-axis represents the hospitalization day, and the *y*-axis represents the scores. A higher number of points indicates a greater nursing demand. The flows between the scores are depicted by curves leading from the source to the target. The thickness of each curve indicates the volume of the flow. The primary patterns observed in this ward are as follows. On day 1, a substantial number of patients had scores ranging from 0 to 2 points, with totals of 613, 608, and 608 in the fiscal years 2017, 2018, and 2019, respectively. From day 1 to day 2, the majority exhibited stable score trajectories: specifically, 446, 435, and 415 patients experienced a score change of 1 point or less in the fiscal years 2017, 2018, and 2019, respectively.

From day 2 to day 3, there was a noticeable rise in patient scores, especially among those whose scores shifted from 0 to between 6 and 9 points, numbering 154, 173, and 206 in the fiscal years 2017, 2018, and 2019, respectively. From day 3 to day 4, these patients' scores stabilized, specifically transitioning from between 6 and 9 points to between 6 and 8 points, with 232, 257, and 267 patients following these trajectories in the fiscal years 2017, 2018, and 2019, respectively. From day 4 to day 5, a declining trend was observed. In particular, 145, 128, and 136 patients with scores between 6 and 8 points saw their scores fall around 4 or 5 points in the fiscal years 2017, 2018, and 2019, respectively.

Beyond day 5, score changes from one day to the next were insignificant. For example, from day 5 to day 6, the scores of 385, 393, and 393 patients remained unchanged in the fiscal years 2017, 2018, and 2019, respectively. Moreover, from day 11 to day 12, the scores of 398, 427, and 401 patients did not change.

In the fiscal year 2017, there were instances of patients following an increasing trajectory, with scores rising from 7 to 10 points between day 9 and day 10, and from 10 to 11 points between day 10 and day 11. Subsequently, for several days, the score remained constant, settling at a high value. In fiscal year 2018, patient trajectories were seen to surpass 10 points between day 3 and day 12. In fiscal year 2019, some patients exhibited an increasing trajectory, with scores rising from 7 to 10 points between day 10 and day 11. Following this surge, the scores plateaued and remained unchanged for several days.

#### 3.1.2. The Neurosurgery Ward

**Figure 4:**
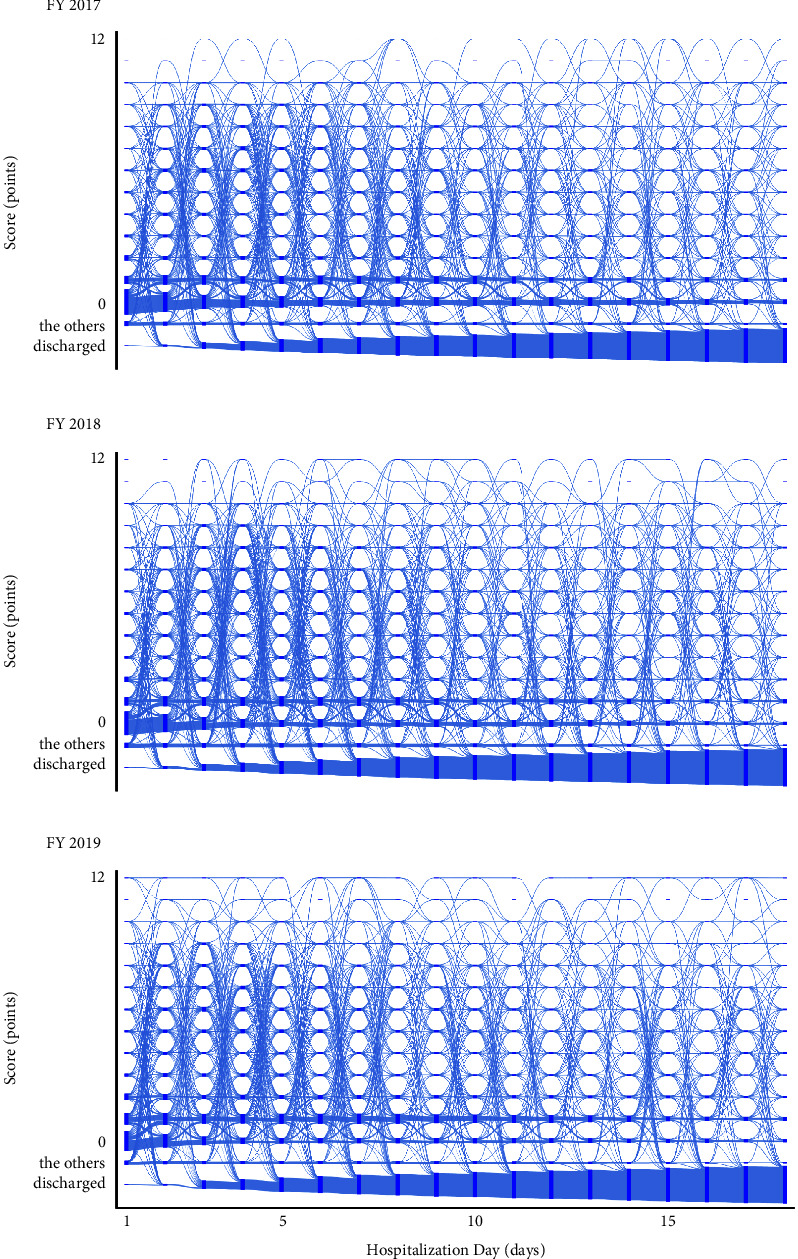
The trajectories of nursing demand in the Neurosurgery Ward for fiscal year (a) 2017, (b) 2018, and (c) 2019. Nodes are represented by rectangles indicating the score. The flows between them are depicted by curves leading from the source to the target nodes. The volume of each flow is indicated by the thickness of the curve. “The others” indicates “staying in other wards or specialized beds.”

All trajectories of the Neurosurgery Ward are shown in Figure 4. The primary patterns observed in this ward are as follows. Scores of 0 or 1 point were consistent throughout the hospitalization period, suggesting that patients who scored 0 or 1 point on a certain day were more likely to maintain similar scores the next day. Particularly, from day 1 to day 2, 412, 414, and 410 patients followed such trajectories in the fiscal years 2017, 2018, and 2019, respectively. Furthermore, from day 2 to day 3, the numbers of patients with these trajectories were 303, 283, and 257 in the fiscal years 2017, 2018, and 2019, respectively. The remaining patients exhibited diverse trajectories; their progression was intricate and multifaceted.

During the hospitalization period, trajectories reaching scores between 10 and 12 points were observed daily. From day 1 to day 2, the numbers of patients experiencing these trajectories were 8, 11, and 15 in the fiscal years 2017, 2018, and 2019, respectively. From day 10 to day 11, the numbers of patients were 10, 13, and 9 in the fiscal years 2017, 2018, and 2019, respectively. From day 17 to day 18, 19, 11, and 8 patients experienced these trajectories in the fiscal years 2017, 2018, and 2019, respectively.

Notably, we observed trajectories that led from high scores, such as between 9 and 12, to “discharged.” During the hospitalization period, a total of 10, 16, and 24 patients experienced these trajectories in the fiscal years 2017, 2018, and 2019, respectively. We also observed trajectories from “staying in other wards or specialized beds” to high scores, between 9 and 12. From day 1 to day 2, the numbers of patients following these trajectories were 6, 5, and 8 in the fiscal years 2017, 2018, and 2019, respectively. From day 13 to day 14, these numbers were 4, 2, and 3 in the fiscal years 2017, 2018, and 2019, respectively. Although detailed numbers are provided for only a few days, this phenomenon was consistent, regardless of the day of admission during hospitalization.

#### 3.1.3. The Cardiovascular Medicine Ward

**Figure 5:**
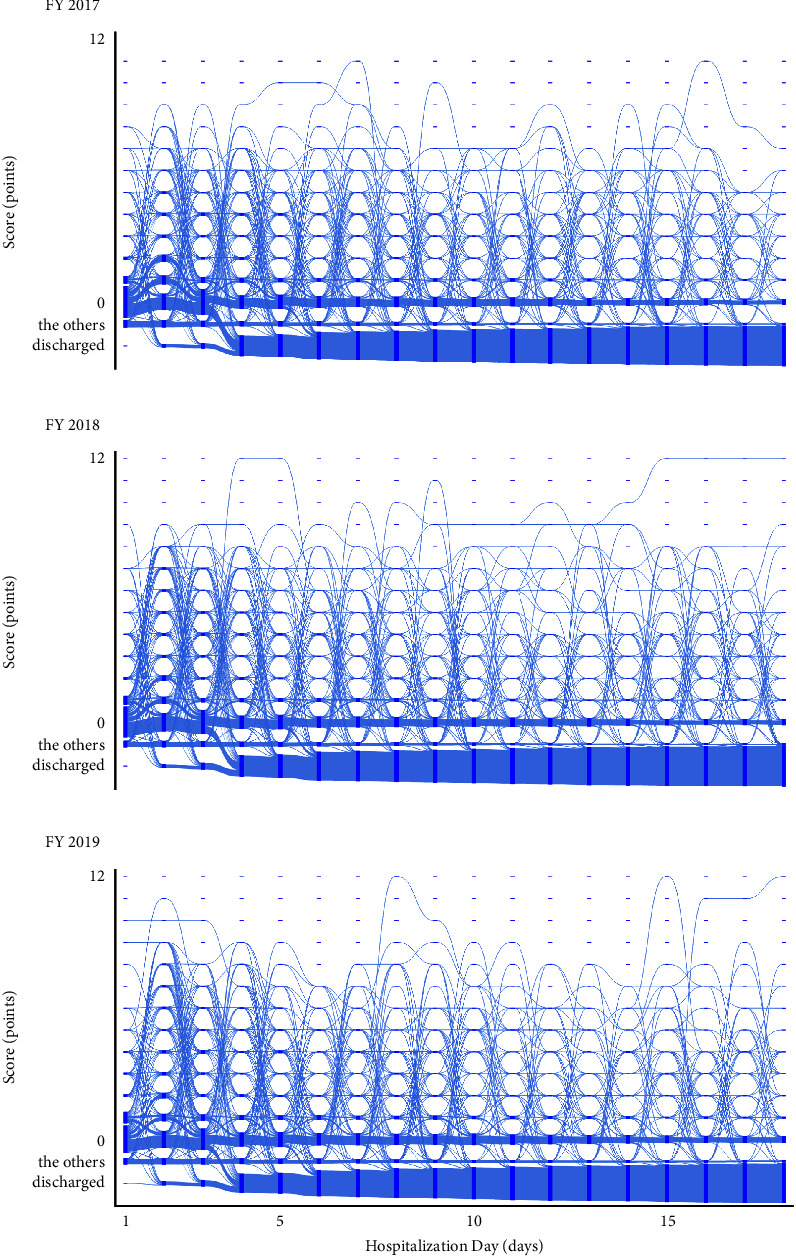
The trajectories of nursing demand in the Cardiovascular Medicine Ward for fiscal year (a) 2017, (b) 2018, and (c) 2019. Nodes are represented by rectangles indicating the score. The flows between them are depicted by curves leading from the source to the target nodes. The volume of each flow is indicated by the thickness of the curve. “The others” indicates “staying in other wards or specialized beds.”

All trajectories of the Cardiovascular Medicine Ward are shown in Figure 5. The primary patterns observed in this ward are as follows. On the first day, many patients (609, 594, and 478 in the fiscal years 2017, 2018, and 2019, respectively) scored 0 points. Approximately half of these patients followed a slightly ascending trajectory, with scores increasing to 1 or 2 points from day 1 to day 2. Specifically, the numbers were 243, 170, and 131 in the fiscal years 2017, 2018, and 2019, respectively. These patients then exhibited a downward trend, with their scores decreasing to 0 from day 2 to day 3, with patient counts of 209, 144, and 108 in the fiscal years 2017, 2018, and 2019, respectively.

A significant number of patients demonstrated patterns leading to “discharge” from day 3 to day 4 and from day 5 to day 6. From day 3 to day 4, 302, 281, and 220 patients were discharged in the fiscal years 2017, 2018, and 2019, respectively. From day 5 to day 6, the discharge numbers were 95, 107, and 94 in the fiscal years 2017, 2018, and 2019, respectively. We also noted trajectories of patients moving from “staying in other wards or specialized beds” to achieving higher scores, typically between 4 and 7 points. For instance, from day 5 to day 6, 12 patients exhibited these trajectories in each fiscal year.

A small proportion of patients followed near-flat trajectories, maintaining extremely high scores, such as between 10 and 12 points, throughout their hospitalization. For example, such trajectories were observed from day 5 to day 6 in the fiscal year 2017; from day 4 to day 5 and from day 15 to day 18 in the fiscal year 2018; and from day 1 to day 3 and from day 8 to day 9 and from day 16 to day 18 in the fiscal year 2019.

### 3.2. The Ward-Specific Feature of the Statistical Regularities in Temporal Dynamics of Nursing Demand

#### 3.2.1. Trajectories of Scores

**Figure 6:**
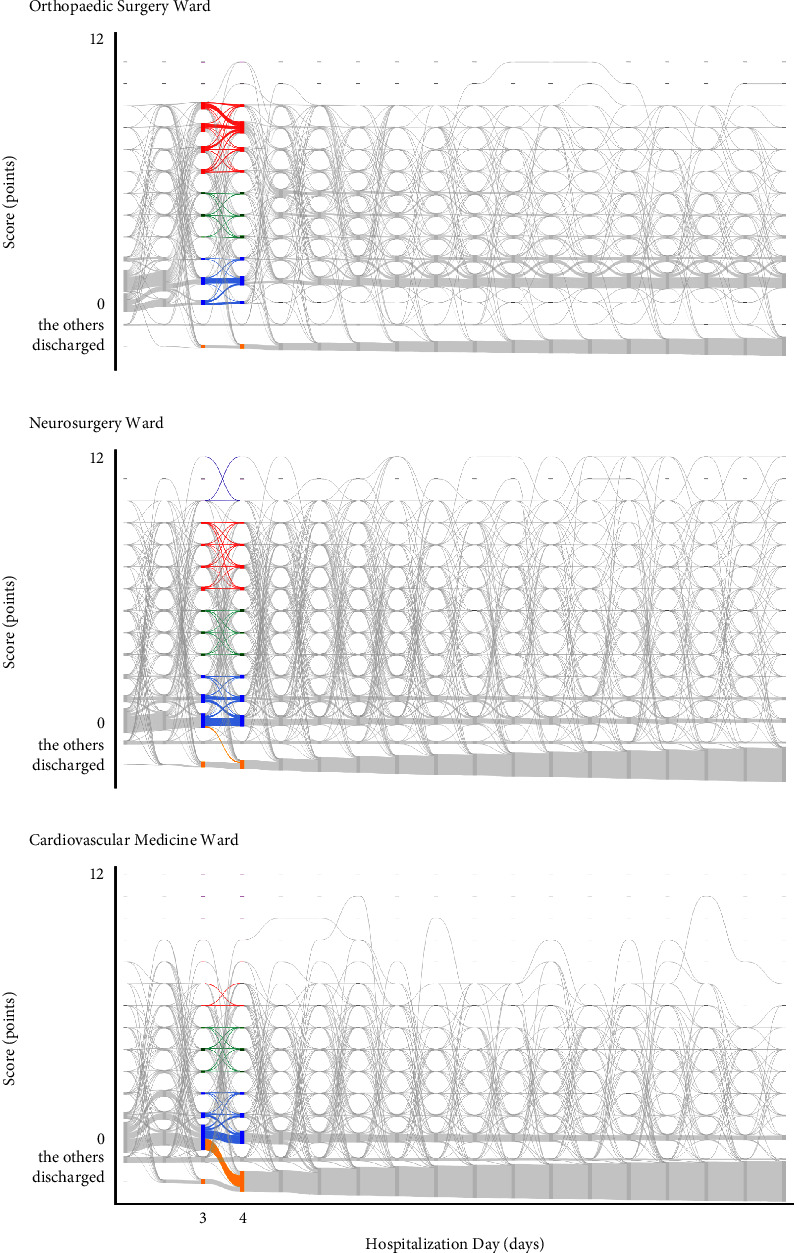
The trajectories of nursing demand classifications from day 3 to day 4 in each ward for fiscal year 2017 (a) in the Orthopaedic Surgery Ward, (b) in the Neurosurgery Ward, and (c) in the Cardiovascular Medicine Ward. The yellow trajectories indicate paths from 0 points to discharge, with blue for low scores, green for medium scores, red for high scores, and purple for ultrahigh scores. Nodes are represented by rectangles indicating the score. The flows between them are depicted by curves leading from the source to the target nodes. The volume of each flow is indicated by the thickness of the curve. “The others” indicates “staying in other wards or specialized beds.”

Here, scores of 0–2 were classified as low, 3–5 as medium, 6–9 as high, and 10–12 as very high. This classification was provisional, used solely to describe the trajectory. This classification was used tentatively only to illustrate the characteristics of the trajectory. Trajectories from day 3 to day 4 were compared for low scores, medium scores, high scores, ultra-high scores, and from 0 to discharge in each ward in 2017. In Figure 6, scores are represented as follows: low scores in blue, medium scores in green, high scores in red, ultra-high scores in purple, and scores from 0 to discharge in orange.

In the Orthopaedic Surgery Ward, the largest group of patients exhibited a trajectory between high scores, with 265 patients (38.7%), followed by trajectories between low scores with 164 patients (24.0%), and medium scores with 27 patients (3.9%). There were no patients transitioning between ultra-high scores or from 0 to discharge. In the Neurosurgery Ward, the most common trajectory was between low scores with 314 patients (37.4%), followed by high scores with 110 patients (13.1%), medium scores with 39 patients (4.6%), from 0 to discharge with 22 patients (2.6%), and ultra-high scores with 8 patients (1.0%). In the Cardiovascular Medicine Ward, the most common trajectory was also between low scores with 325 patients (32.7%), followed by trajectories from 0 to discharge with 218 patients (21.9%), medium scores with 33 patients (3.3%), and high scores with 5 patients (0.5%). There were no patients transitioning between ultra-high scores.

Thus, in the Orthopaedic Surgery Ward, unlike other wards, a high percentage of patients transitioned between high scores. Also, the trajectories were biased towards high and low scores, indicating a distinct division in patient trajectories that was unique to this ward. In the Neurosurgery Ward, similar to the Cardiovascular Medicine Ward, the majority of patients followed the trajectory between low scores, and the proportion of patients was similar. However, trajectories in other scores were almost evenly distributed among patients, and there were patients transitioning between ultra-high scores, which was not observed in other wards. In the Cardiovascular Medicine Ward, like the Neurosurgery Ward, the most common trajectory was between low scores. However, unlike other wards, there was a higher percentage of patients transitioning from 0 to discharge, showing a bias towards trajectories between low scores and from 0 to discharge. In addition, compared to other wards, there were fewer patients transitioning between high scores and ultra-high scores.

#### 3.2.2. Diversity of Trajectories

**Figure 7:**
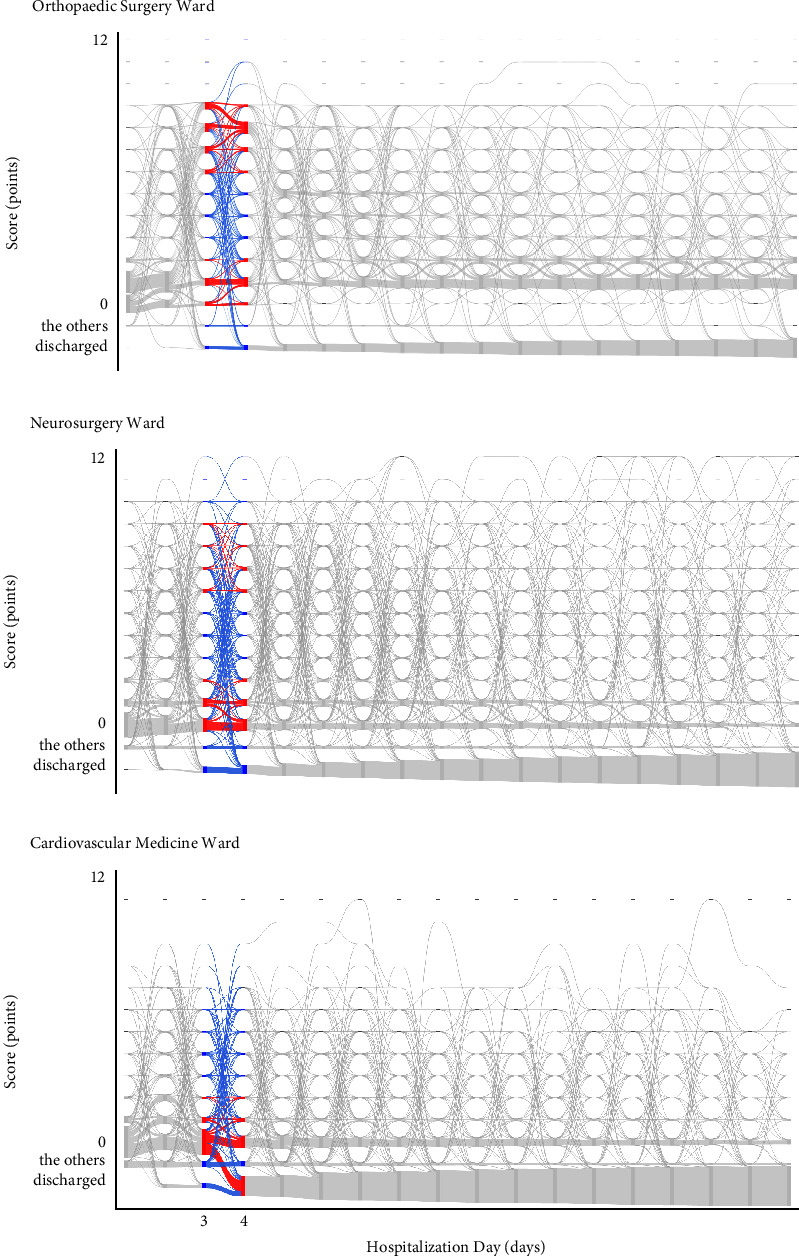
The diversity of nursing demand trajectories from day 3 to day 4 in each ward for fiscal year 2017 (a) in the Orthopaedic Surgery Ward, (b) in the Neurosurgery Ward, and (c) in the Cardiovascular Medicine Ward. The red trajectories represent the two most frequent paths, while the blue trajectories denote all other paths. The flows between them are depicted by curves leading from the source to the target nodes. The volume of each flow is indicated by the thickness of the curve. “The others” indicates “staying in other wards or specialized beds.”

Using the same period and scoring classification, differences in the diversity of trajectories were compared. In Figure 7, the red trajectories represent the two most frequent paths, while the blue trajectories denote all other paths. In the Orthopaedic Surgery Ward, there were 84 trajectories out of 225 possible ones. The scores ranged from 0 to 11 points. 164 patients (24.0%) followed trajectories between low scores, 27 people (3.9%) between medium scores, and 265 people (38.7%) between high scores. No patients transitioned between ultra-high scores or from 0 to discharge. A notable 62.7% of patients were concentrated in the top two scoring layers, indicated in red. In the Neurosurgery Ward, there were 104 trajectories out of 225 possible ones, with scores ranging from 0 to 12 points. 314 people (37.4%) exhibited trajectories between low scores, 39 people (4.6%) between medium scores, 110 people (13.1%) between high scores, 8 people (1.0%) between ultra-high scores, and 22 people (2.6%) from 0 to discharge. A total of 50.5% of patients were concentrated in the top two scoring layers, shown in red. In the Cardiovascular Medicine Ward, there were 73 trajectories out of 225 possible ones, with scores ranging from 0 to 9 points. 325 people (32.7%) showed trajectories between low scores, 33 people (3.3%) between medium scores, 5 people (0.5%) between high scores, and 218 people (21.9%) from 0 to discharge. No patients transitioned between ultra-high scores. A total of 54.6% of patients were concentrated in the two highest-scoring layers, shown in red.

The Orthopaedic Surgery Ward showed a wider range of scores yet had the highest proportion of patients following notable trajectories, indicating not only a large bias towards notable trajectories but also other patients followed relatively limited trajectories. This was reflected in the diagram by thick red trajectories and a wide range of low-density blue trajectories. The Neurosurgery Ward, with a wider range of scores, had the lowest proportion of patients following notable trajectories, showing the most diverse patterns. This indicated a smaller bias towards significant trajectories, with other patients following a wide range of diverse trajectories. This was represented in the diagram by thin red trajectories and a wide range of high-density red trajectories. The Cardiovascular Medicine Ward, despite a moderate proportion of patients following notable trajectories, had the narrowest range of scores and the fewest trajectory patterns. While there were not many patients following notable trajectories, other patients were following specific trajectories within a narrow range. This was represented in the diagram by relatively thick red trajectories and a narrow range of low-density blue trajectories.

While each ward exhibited several prominent trajectories followed by a significant number of patients, we observed noticeable differences among these trajectories across the wards. Consequently, each ward exhibited unique features in the trajectory of nursing demand. As these features indicated, the trajectories of nursing demand were more similar when we compared them between fiscal years in the same wards than when we compared them between wards. Due to space constraints in this paper, we only presented results from the three wards. However, in practice, similar trends were obtained in the other 17 wards where the same indicator was used.

## 4. Discussion

### 4.1. The Statistical Regularities in Temporal Dynamics of Nursing Demand across Fiscal Years within Each Ward

The temporal dynamics of nursing demand are similar from year to year when the first day of each patient's admission is set as the starting point, and the dynamics are viewed from the perspective of the patient population. These similarities suggest the existence of statistical regularities in nursing demand. Clinically, the nursing demand with the progression of hospitalization days in different groups of newly admitted patients may change probabilistically in the same manner.

These statistical regularities could, in part, be attributable to the decisions made regarding treatment and the support given to patients to follow the course of treatment according to their clinical pathways. In clinical practice sites, when treatment methods are determined, patients are considered to follow a certain predictable course, enabling the provision of nursing care to be aligned with the clinical pathways, excluding patients with multiple comorbidities, dementia, or advanced age. In addition, patients managed with clinical pathways exhibit higher protocol adherence and lower incidence of complications than those without clinical pathways [31, 32]. Thus, the decisions made regarding treatment and the implementation of the clinical pathways could potentially contribute to the similar nursing demand among the patient population in each ward.

The statistical regularities in nursing demand are also considered to be attributable to the biological healing process following a specific course. This is because, despite the clinical pathway compliance rate in the target hospital being less than 50%, more than half of the inpatients appeared to follow a similar trend. When a living body undergoes an invasive treatment, including surgical intervention, it triggers a stress response, striving to maintain homeostasis [33]. Furthermore, postintervention, the body recovers by passing through stages of “injury,” “the turning point,” “muscular strength,” and “fat gain” [34]. Thus, the somewhat rule-based recovery process of a body following an invasive procedure could contribute to the regularities observed in temporal dynamics of nursing demand.

The existence of statistical regularities potentially indicates that we may be able to probabilistically forecast the temporal variation of nursing demand among patients in future years by utilizing historical data on nursing demand. That is because patterns of temporal dynamics of nursing demand are not coincidental but inherent characteristics of the respective wards, and the same phenomena could potentially occur every year. In essence, by creating a system that predicts temporal variations based on past nursing demand data, we may be able to forecast individual patients' temporal changes in nursing demand when this system is applied to future data.

Predicting nursing demand based on statistical regularities will enable the simulation of efficient staff allocation by determining which patients should be assigned to which nurses, ultimately ensuring patient safety with fewer nurses. Although, some prior studies have estimated the number of nurses needed for patient populations, there is a lack of evidence regarding the assignment of individual nurses to individual patients. The nursing shortage was reported to be resolved by securing a sufficient baseline number of full-time nurses and addressing any deficiencies with the addition of temporary staff [35]. However, increasing the number of nurses becomes progressively more difficult due to the looming nursing shortage. Even with additional temporary nurses, a lack of coordination among staff may leave the care undone, threatening patient safety [36]. Thus, we should assign the limited full-time nursing resources available to patients more efficiently. The inherent statistical regularities in nursing demand can predict changes in nursing demand and inform a simulation model. This model can calculate optimal solutions for assigning nurses to patients [37], thus facilitating the determination of efficient nurse assignments to meet the daily changing needs of individual patients. However, it should be noted that this application assumes equality in nurses' factors. When assigning individual nurses to individual patients, we must consider the nurses' abilities [38] and their perceived workload [39]. To apply these regularities to a staffing model, further examination of additional factors is necessary.

Our use of extensive data represents one of the significant strengths of this study. This allowed us to identify the full range of trajectories in the target wards and to investigate potential statistical regularities. In addition, our analysis was not affected by seasonality since we did not focus on a specific short-term period. Analysis of all patients admitted to the target wards of the target hospital over a three-year period, excluding those with missing data, greatly facilitated our ability to attain these results.

We observed a certain number of patients experiencing a sudden surge in scores or consistently transitioning with high scores each year, especially in the Orthopaedic Surgery Ward and the Cardiovascular Medicine Ward. This suggests that there are also statistical regularities among a small number of patients within the population. These patients had been likely to develop complications or exacerbate comorbidities. That is because, while the proportions vary, some patients have experienced variances in their clinical pathways [40, 41]. In addition, the occurrence of postoperative complications has been associated with an increase in nursing workload, that is, the increase in nursing demand [42]. Thus, the occurrence of complications is considered to result in increases in nursing demand. However, it remains unclear whether there is any regularity in the timing of these occurrences or the scores assigned to them. Future research should aim to elucidate these uncertainties and investigate the features of temporal changes in abnormal patients. This is crucial because, despite the challenges in forecasting sudden changes and abnormal recovery processes from an individual perspective, such occurrences necessitate a substantial amount of nursing care, which can place a significant burden on nurses [42]. To provide necessary care to these patients, as well as the broader patient population, it is essential to adjust nursing schedules and devote nursing resources. The ability to forecast patients with a rapid increase in nursing demand is thought to facilitate efficient, effective, and flexible nursing staff scheduling. In order to adequately devote nursing resources to address the occurrence of abnormal patients, it is necessary to investigate the statistical regularities in their temporal changes.

In the Neurosurgery Ward, it was not possible to clearly discern patients who were considered to experience complications and therefore to undergo a rapid change in nursing demand. The reason is that the patient population in this ward exhibited a greater variety of nursing demand change patterns compared to the other two wards. Each patient followed various trajectories, meaning that the proportion of patients following each trajectory is low, or in other words, the “flow” is “thin.” Consequently, the trajectories of such a small number of patients may have been masked.

### 4.2. The Ward-Specific Feature of the Statistical Regularities in Temporal Dynamics of Nursing Demand

The statistical regularity inherent in the trajectories of nursing demand is evidently ward-specific. This may be attributed to the different types or levels of nursing care required in different wards, as diverse diseases and treatments impair varying functions in patients [43, 44]. For instance, in the Orthopaedic Surgery Ward, nurses often need to provide considerable assistance to mobilize patients due to their physical dysfunction. A significant amount of care is especially needed just after surgery, and the level of care typically decreases as the days of hospitalization progress. Conversely, in the Neurosurgery Ward, they must manage risk behaviors caused by patients' cognitive dysfunction. The causes of cognitive impairments, such as delirium, often occur suddenly. Moreover, in the Cardiovascular Medicine Ward, nurses often offer various types of care related to ADL since patients often struggle to perform these tasks independently due to circulatory instability or fatigue throughout their hospital stay. Consequently, it is plausible that the specific nature of diseases and treatments gives rise to ward-specific regularities in the temporal dynamics of nursing demand. However, we did not identify the differences in diseases and treatments among participants. Therefore, the detailed causes of these variations in dynamics are speculative at best. Future research needs to pinpoint the factors contributing to the statistical regularities in temporal dynamics, as the amount and timing of nursing care needed to meet the demands of the patient population are likely dependent on its components.

It is essential to forecast the temporal changes in nursing demand on a ward-by-ward basis for nursing management. Indeed, wards serve as the fundamental units of nursing management, with nursing staff scheduling and bed management often implemented at this level. The ward-specific statistical regularities can offer valuable insights for managing nursing in practical settings.

The ward-specific statistical regularities can extend beyond utilization within the ward itself, potentially applying to staff allocations from the hospital to individual wards. By predicting and aggregating nursing demand for each ward, it is possible to estimate the range of basic nursing volume required by patient populations in each ward [45]. The estimated overall nursing demand can lead to estimations of the fundamental number of nurses needed for each ward [45, 46]. Furthermore, wards experiencing significant fluctuations in overall nursing demand may require more flexible staff allocations. Aggregation by ward can also inform the examination of the ratio between fixed and temporary staff numbers. This approach serves as one process in determining the number of nurse staff to be allocated from the hospital to each ward, representing the utilization of nursing management from a more macroperspective than daily assignments. As previously mentioned, this application assumes equality in nurses' factors, such as abilities and perceived workload. Further research is necessary to identify the factors that should be considered in developing a staffing model.

Since various factors influence the nature of nursing demand [37], charge nurses and nurse managers, who focus more on administrative and managerial duties, have to devote daily nursing resources and permanent nurses based on the nursing demand forecasted from various perspectives. This study represents one such perspective necessary for forecasting nursing demand. Our findings are expected to contribute to forecasting nursing demand and estimating nursing resources, leading to data-driven, more efficient nursing management.

## 5. Conclusions

The main finding of this study is that the temporal changes in nursing demand demonstrate greater similarity when compared across fiscal years within individual wards than when compared between different wards. This observation suggests the existence of statistical regularity in the temporal changes in nursing demand within each ward. Furthermore, it implies that the temporal changes in nursing demand exhibit unique statistical regularities in each different ward.

As demonstrated in this paper, the statistical regularity is intuitively identified by viewing the temporal changes in nursing demand within each ward as an overlay of multiple trajectories. In addition, we were able to distill statistical regularities across multiple fiscal years by harnessing a substantial amount of data, by encapsulating the influence of individual patients within these regularities.

These findings suggest that patients admitted to the wards are considered to exhibit a certain probabilistic change in nursing demand. It can be inferred that the temporal changes in nursing demand of the patient population are potentially predicted probabilistically, which is useful for nursing management.

## 6. Limitation

This study has four limitations.

First, the inherent statistical regularities presuppose that the medical and nursing systems have not undergone significant changes over the fiscal years. In fact, a period of three years without such changes was selected for analysis. Specifically, data were extracted from a period without significant ward reorganization due to the COVID-19 pandemic. Changes in hospital operations, such as altering the patient-to-nurse ratio or patient characteristics, may result in a failure to recognize the statistical regularities in nursing demand. Consequently, additional Sankey diagrams may be required to identify new statistical regularities. After the nature of the patterns has changed, within a period where the situation remains unchanged, it will be possible to determine certain regularities, as mentioned in this study.

Second, although the data were thoroughly utilized for the management of the target hospital, the results may lack generalizability as they were collected from a single acute hospital. The results may not be directly applicable to hospitals or facilities of different sizes or service offerings, particularly smaller healthcare facilities or those specializing in specific medical services. To address this, it would be beneficial to analyze data from patient populations admitted to each hospital in a similar manner. For example, a secondary analysis of data independently recorded at each hospital or facility can identify patterns of nursing demand inherent in the patient populations of those facilities.

Third, the score for item “B” in the INCN only quantifies certain aspects of nursing demand related to ADL and cognition. As such, it may not encapsulate the entirety of nursing demand. However, a statistical regularity is presumed to exist in the temporal dynamics of nursing demand when using this particular indicator.

Last, the score for item “B” in the INCN might not fully represent all nursing demands related to ADL and cognition encapsulated within item “B.” This is because these scores are determined based on the actual provision of nursing care. In situations where patients require nursing care from a professional nursing perspective, but such care is not provided, no points are added to the score. Therefore, if the score truly represented nursing demand, the similarity of temporal dynamics between fiscal years might decrease.

## 7. Implications for Nursing Management

In this study, we identified ward-specific statistical regularities in the temporal dynamics of nursing demand. The existence of such regularities suggests the possibility of forecasting changes in nursing demand and strategizing accordingly [47]. Specifically, we could potentially forecast temporal changes in nursing demand by utilizing data from past fiscal years. This would enable charge nurses and nurse managers to staff efficiently from two main perspectives. First, on a ward-by-ward basis, charge nurses can assign individual nurses to patients daily more efficiently based on data. For example, an experienced nurse might be assigned to a patient for whom dynamic demand changes are expected; conversely, an inexperienced nurse might be assigned to a patient for whom few demand changes are expected. Charge nurses could assign available fixed nurses to patients to ensure patient safety even in situations where the nursing shortage makes it difficult to secure adequate staffing [36]. Second, in management from the hospital to each ward, by predicting and aggregating nursing demand for each ward, nurse managers can estimate the range of basic nursing volume required by the patient population [45]. They can adjust the ratio between fixed and temporary staff to meet these needs effectively. For instance, they should assign more fixed nurses in wards with small fluctuations in nursing demand and, conversely, more temporary nurses in wards with large fluctuations in nursing demand. Ward-specific patterns could also be utilized for hospital-wide management.

In various countries, it is possible to analyze data using the nursing demand indicators relevant to each nation. For instance, various countries employ the NAS to measure direct and indirect nursing activities [10]. In Finland, the OPC instrument is used to assess the quantity and level of nursing care provided to individual patients [11, 12]. We anticipate that the identification of statistical regularities in nursing demand, as demonstrated in this study, will contribute to more efficient nursing management in various countries.

We strongly advocate for charge nurses and nurse managers to leverage the secondary analysis of extensive data. In the field of nursing practice, a variety of indicators are preserved as large volumes of data and used for different purposes. However, they are primarily employed only within the scope of their original intent. More meticulous management is necessary to ensure the adequate provision of various types and levels of nursing care, avoiding overworking nurses or straining hospital finances, especially in anticipation of future nursing resource shortages [5]. Nursing management is often executed based on assumptions and intuition derived from experienced experts such as chief nurses. However, it is crucial to base forecasts of nursing demand and estimations of necessary nursing resources on data analysis. Relying solely on expert insight could result in a decline in management quality in their absence. Given the aging and declining nursing population, concerns arise about potential future shortages of such experts. Data-driven forecasts and estimations allow nurses of all levels of experience to implement nursing management strategies with the same efficacy, regardless of their individual expertise. Future research should explore the combination of various existing data to implement data-driven nursing management, contributing to the development of nursing sciences.

## Figures and Tables

**Table 1:** Criteria for the score for item “B” in the intensity of nursing care needs.

System	Score		
	0	1	2
Rolling overact	Can do it	Can do it if holding onto something	Cannot do it
Transferring	Independent	Partial assistance	Full assistance
Oral health	Independent	Assistance	—
Dietary intake	Independent	Partial assistance	Full assistance
Changing clothes	Independent	Partial assistance	Full assistance
Instruction for medical care and treatment	Compliance with instruction	Noncompliance with instruction	—
Risk behavior	Avoid risk behavior	—	Display risk behavior

**Table 2:** Descriptive characteristics of the patients.

	Orthopaedic Surgery Ward			Neurosurgery Ward			Cardiovascular Medicine Ward		
	2017	2018	2019	2017	2018	2019	2017	2018	2019
*Patient, number*									
All	684	697	706	840	879	967	1000	1006	899
Eligible	684	688	691	839	873	959	994	998	898
Excluded	0	9	15	1	6	8	6	8	1

Age, mean (SD)	54.2 (20.2)	54.6 (20.8)	56.1 (20.8)	57.4 (17.9)	57.3 (18.3)	57.7 (18.3)	65.0 (16.9)	65.8 (16.0)	66.0 (15.8)

*Sex, number (%)*									
Male	257 (37.6)	261 (37.9)	266 (38.5)	446 (53.2)	456 (52.2)	454 (47.3)	637 (64.1)	688 (68.9)	582 (64.8)
Female	427 (62.4)	427 (62.1)	425 (61.5)	393 (46.8)	417 (47.8)	505 (52.7)	357 (35.9)	310 (31.1)	316 (35.2)

Score on day 1, median (Q1–Q3)	1.0 (0.0–1.0)	1.0 (0.0–1.0)	1.0 (0.0–1.0)	0.0 (0.0–2.0)	1.0 (0.0–3.0)	1.0 (0.0–3.0)	0.0 (0.0–1.0)	0.0 (0.0–1.0)	0.0 (0.0–1.0)

SD, standard error; Q1, first quartile; Q3, third quartile.

**Table 3:** Temporal changes in inpatient numbers and scores.

	Orthopaedic Surgery Ward					
	2017		2018		2019	
	Inpatients, number (%)	Score, median (Q1–Q3)	Inpatients, number (%)	Score, median (Q1–Q3)	Inpatients, number (%)	Score, median (Q1–Q3)

*Day*						
1	684 (100.0)	1.0 (0.0–1.0)	688 (100.0)	1.0 (0.0–1.0)	691 (100.0)	1.0 (0.0–1.0)
10	526 (76.9)	2.0 (1.0–4.0)	517 (75.1)	2.0 (1.0–3.0)	495 (71.6)	2.0 (1.0–4.0)
18	421 (61.5)	2.0 (1.0–3.0)	410 (59.6)	1.0 (1.0–3.0)	342 (49.5)	1.0 (1.0–3.0)

	Neurosurgery Ward					
	2017		2018		2019	
	Inpatients, number (%)	Score, median (Q1–Q3)	Inpatients, number (%)	Score, median (Q1–Q3)	Inpatients, number (%)	Score, median (Q1–Q3)

*Day*						
1	839 (100.0)	0.0 (0.0–2.0)	873 (100.0)	1.0 (0.0–3.0)	959 (100.0)	1.0 (0.0–3.0)
10	477 (56.9)	1.0 (0.0–4.0)	457 (52.3)	1.0 (0.0–5.0)	497 (51.8)	1.0 (1.0–5.0)
18	264 (31.5)	1.0 (0.0–6.0)	238 (27.3)	2.0 (1.0–6.0)	261 (27.2)	2.0 (1.0–5.0)

	Cardiovascular Medicine Ward					
	2017		2018		2019	
	Inpatients, number (%)	Score, median (Q1–Q3)	Inpatients, number (%)	Score, median (Q1–Q3)	Inpatients, number (%)	Score, median (Q1–Q3)

*Day*						
1	994 (100.0)	0.0 (0.0–1.0)	998 (100.0)	0.0 (0.0–1.0)	898 (100.0)	0.0 (0.0–1.0)
10	348 (35.0)	0.0 (0.0–2.0)	331 (33.2)	0.0 (0.0–3.0)	342 (38.1)	0.0 (0.0–2.0)
18	207 (20.8)	1.0 (0.0–2.0)	203 (20.3)	0.0 (0.0–2.0)	203 (22.6)	0.0 (0.0–2.0)

Q1, first quartile; Q3, third quartile.

## Data Availability

The access data used to support the findings of this study have not been made available to protect personal information.

## References

[1] Fagerström L., Kinnunen M., Saarela J. (2018). Nursing workload, patient safety incidents and mortality: an observational study from Finland. *BMJ Open*.

[2] Aiken L. H., Clarke S. P., Sloane D. M., Sochalski J., Silber J. H. (2002). Hospital nurse staffing and patient mortality, nurse burnout, and job dissatisfaction. *JAMA*.

[3] Aiken L. H., Sloane D. M., Bruyneel L. (2014). Nurse staffing and education and hospital mortality in nine European countries: a retrospective observational study. *The Lancet*.

[4] Lasater K. B., Aiken L. H., Sloane D. (2021). Patient outcomes and cost savings associated with hospital safe nurse staffing legislation: an observational study. *BMJ Open*.

[5] Park C. S. Y. (2023). More is not always better: park’s sweet spot theory-driven implementation strategy for viable optimal safe nurse staffing policy in practice. *International Nursing Review*.

[6] Park C. S. Y. (2017). Optimizing staffing, quality, and cost in home healthcare nursing: theory synthesis. *Journal of Advanced Nursing*.

[7] Park C. S. Y. (2018). Challenging rules, creating values: park’s sweet spot theory-driven Central-Optimum nurse staffing zone. *Journal of Advanced Nursing*.

[8] Pitkäaho T., Partanen P., Miettinen M., Vehviläinen-Julkunen K. (2015). Non-linear relationships between nurse staffing and patients’ length of stay in acute care units: bayesian dependence modelling. *Journal of Advanced Nursing*.

[9] Reis Miranda D., Nap R., De Rijk A. (2003). Nursing activities score. *Critical Care Medicine*.

[10] Carmona-Monge F. J., Rollán Rodríguez G. M., Quirós Herranz C., García Gómez S., Marín-Morales D. (2013). Evaluation of the nursing workload through the nine equivalents for nursing manpower use scale and the nursing activities score: a prospective correlation study. *Intensive and Critical Care Nursing*.

[11] Fagerström L., Rainio A. K. (1999). Professional assessment of optimal nursing care intensity level: a new method of assessing personnel resources for nursing care. *Journal of Clinical Nursing*.

[12] Fagerström L., Rainio A. K., Rauhala A., Nojonen K. (2000). Validation of a new method for patient classification, the Oulu patient classification. *Journal of Advanced Nursing*.

[13] Decock K., Casaer M. P., Guïza F. (2020). Predicting patient nurse-level intensity for a subsequent shift in the intensive care unit: a single-centre prospective observational study. *International Journal of Nursing Studies*.

[14] Romano J. L., Garcia P. C., Silva D. V., Moura B. R. S., de Souza Nogueira L. (2019). Type of admission and nursing workload of critical patients: a cross-sectional study. *Nursing in Critical Care*.

[15] De Souza Nogueira L., De Alencar Domingues C., Poggetti R. S., De Sousa R. M. C. (2014). Nursing workload in intensive care unit trauma patients: analysis of associated factors. *PLoS One*.

[16] Padilha K. G., de Sousa R. M. C., Queijo A. F., Mendes A. M., Miranda D. R. (2008). Nursing activities score in the intensive care unit: analysis of the related factors. *Intensive and Critical Care Nursing*.

[17] Shiryaev A. N. (1996). *Probability*.

[18] Menezes K. V. R. S., Auger C., Barbosa J. F. S., Gomes C. S., Menezes W. R. S., Guerra R. O. (2021). Trajectories and predictors of functional capacity decline in older adults from a Brazilian northeastern hospital. *Journal of Geriatric Physical Therapy*.

[19] Feng C. K., Chen M. L., Mao I. F. (2007). Prevalence of and risk factors for different measures of low back pain among female nursing aides in Taiwanese nursing homes. *BMC Musculoskeletal Disorders*.

[20] Smedley J., Egger P., Cooper C., Coggon D. (1997). Prospective cohort study of predictors of incident low back pain in nurses. *BMJ*.

[21] Griffiths P., Saville C., Ball J. E., Jones J., Monks T. (2021). Beyond ratios-flexible and resilient nurse staffing options to deliver cost-effective hospital care and address staff shortages: a simulation and economic modelling study. *International Journal of Nursing Studies*.

[22] Schäfer F., Walther M., Hübner A., Kuhn H. (2019). Operational patient-bed assignment problem in large hospital settings including overflow and uncertainty management. *Flexible Services and Manufacturing Journal*.

[23] Soh J. Y., Jung S. H., Cha W. C. (2019). Variability in doctors’ usage paths of mobile electronic health records across specialties: comprehensive analysis of log data. *JMIR Mhealth Uhealth*.

[24] Carleton N., Zou J., Fang Y. (2021). Outcomes after sentinel lymph node biopsy and radiotherapy in older women with early-stage, estrogen receptor-positive breast cancer. *JAMA Network Open*.

[25] Python Software Foundation (2023). Python 3.11.7.

[26] Pandas development team (2023). What’s New in pandas 2.1.1.

[27] NumPy developers (2023). NumPy v1.25.0 release notes.

[28] Plotly (2023). Sankey diagram.

[29] Plotly (2023). Release v5.15.0.

[30] Andriollo E., Caimo A., Secco L., Pisani E. (2021). Collaborations in environmental initiatives for an effective “adaptive governance” of social–ecological systems: what existing literature suggests. *Sustainability*.

[31] van der Kolk M., van den Boogaard M., Becking-Verhaar F. (2017). Implementation and evaluation of a clinical pathway for pancreaticoduodenectomy procedures: a prospective cohort study. *Journal of Gastrointestinal Surgery*.

[32] Mohamed W. R. A., Leach M. J., Reda N. A., Abd-Ellatif M. M., Mohammed M. A., Abd-Elaziz M. A. (2018). The effectiveness of clinical pathway-directed care on hospitalisation-related outcomes in patients with severe traumatic brain injury: a quasi-experimental study. *Journal of Clinical Nursing*.

[33] Desborough J. P. (2000). The stress response to trauma and surgery. *British Journal of Anaesthesia*.

[34] Francis D. (1959). Moore metabolic care of the surgical patient.

[35] Saville C., Monks T., Griffiths P., Ball J. E. (2021). Costs and consequences of using average demand to plan baseline nurse staffing levels: a computer simulation study. *BMJ Quality and Safety*.

[36] Senek M., Robertson S., Ryan T., King R., Wood E., Tod A. (2020). The association between care left undone and temporary nursing staff ratios in acute settings: a cross- sectional survey of registered nurses. *BMC Health Services Research*.

[37] Ishii A., Nakamura T., Ohno Y., Kasahar S. (2012). Investigation of a methodology for the quantitative estimation of nursing tasks on the basis of time study data. *Advances in Discrete Time Systems*.

[38] Choi J. S., Staggs V. S. (2014). Comparability of nurse staffing measures in examining the relationship between rn staffing and unit-acquired pressure ulcers: a unit-level descriptive, correlational study. *International Journal of Nursing Studies*.

[39] Sir M. Y., Dundar B., Barker Steege L. M., Pasupathy K. S. (2015). Nurse-patient assignment models considering patient acuity metrics and nurses’ perceived workload. *Journal of Biomedical Informatics*.

[40] Shoji F., Yano T., Haro A. (2011). Assessing a clinical pathway to improve the quality of care in pulmonary resections. *Surgery Today*.

[41] Hirao M., Tsujinaka T., Takeno A., Fujitani K., Kurata M. (2005). Patient-controlled dietary schedule improves clinical outcome after gastrectomy for gastric cancer. *World Journal of Surgery*.

[42] Cohen M. M., O’brien-Pallas L. L., Copplestone C., Wall R., Porter J., Rose D. K. (1999). Nursing workload associated with adverse events in the postanesthesia care unit. *Anesthesiology*.

[43] Meyer K. R., Fraser P. B., Emeny R. T. (2020). Development of a nursing assignment tool using workload acuity scores. *The Journal of Nursing Administration: The Journal of Nursing Administration*.

[44] Ko Y., Park B. (2023). Calculating the optimal number of nurses based on nursing intensity by patient classification groups in general units in South Korea: a cross-sectional study. *Nursing Open*.

[45] Padilha K. G., de Sousa R. M. C., Garcia P. C., Bento S. T., Finardi E. M., Hatarashi R. H. K. (2010). Nursing workload and staff allocation in an intensive care unit: a pilot study according to nursing activities score (NAS). *Intensive and Critical Care Nursing*.

[46] Junttila J. K., Koivu A., Fagerström L., Haatainen K., Nykänen P. (2016). Hospital mortality and optimality of nursing workload: a study on the predictive validity of the rafaela nursing intensity and staffing system. *International Journal of Nursing Studies*.

[47] Fukushige H., Ishii A., Inoue Y. (2021). Identifying periodicity in nurse call occurrence: analysing nurse call logs to obtain information for data-based nursing management. *Journal of Nursing Management*.

